# Correction: Multimerization strategies for efficient production and purification of highly active synthetic cytokine receptor ligands

**DOI:** 10.1371/journal.pone.0238925

**Published:** 2020-09-03

**Authors:** Sofie Mossner, Hoang T. Phan, Saskia Triller, Jens M. Moll, Udo Conrad, Jürgen Scheller

## Notice of republication

Incorrect versions of Figs 1, 2, 3, 4, 5, S3 and S4 were published in error. This article was republished on August 6, 2020, to correct for this error. Please download this article again to view the correct version.
