# Supplementary figures and images for: Multimerization strategies for efficient production and purification of highly active synthetic cytokine receptor ligands
Source: PLoS One. 2020 Apr 1;15(4):e0230804. doi: 10.1371/journal.pone.0230804 (PMC7112226; doi:10.1371/journal.pone.0230804)

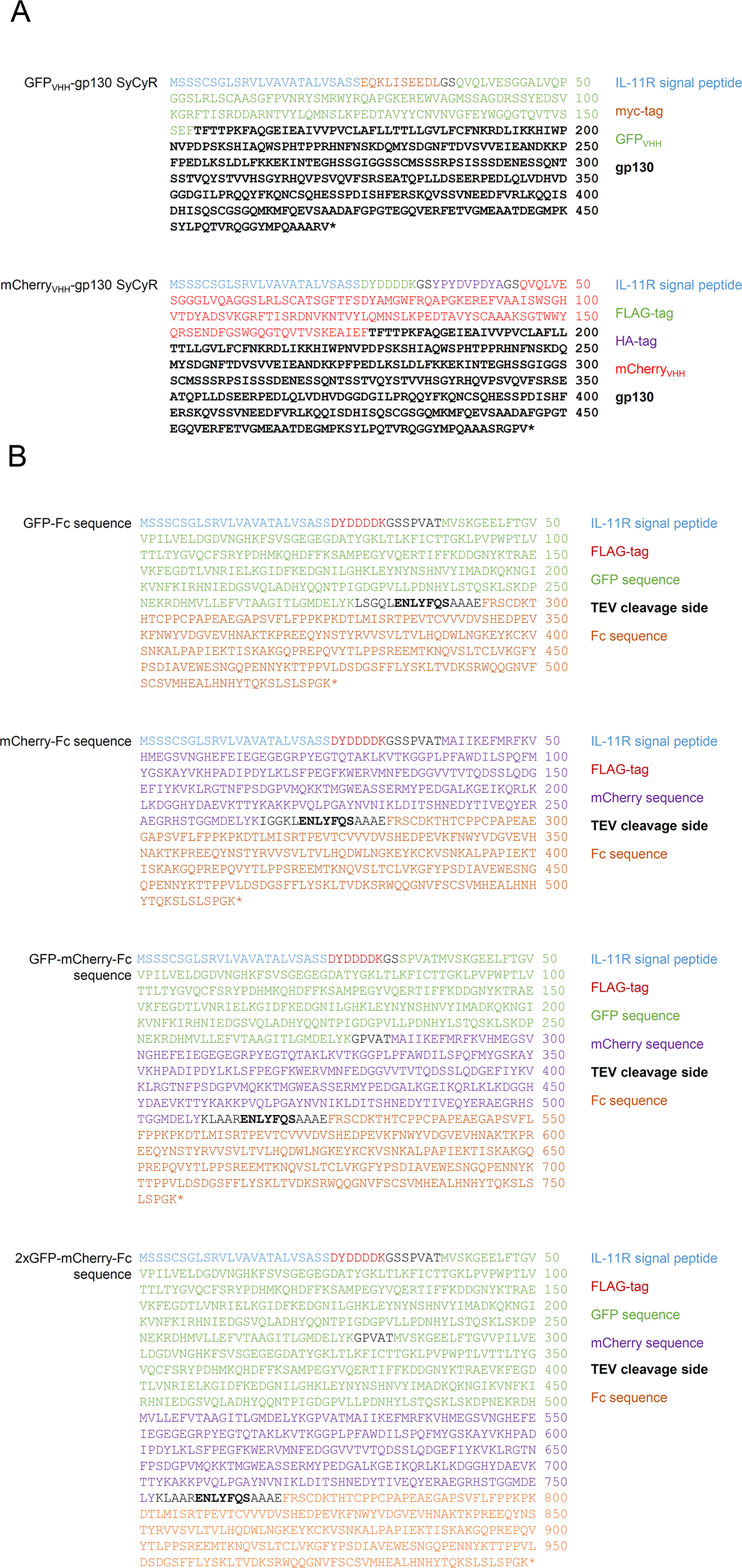

Supplement: S1 Fig — (A) Amino acid sequences of synthetic GFPVHH- and mCherryVHH-gp130 SyCyRs. From N- to C-terminus GFPVHH-gp130: signal peptide of IL-11R (blue), myc-tag (orange), GFPVHH (green) and 13 aa of the ECD, TM and ICD gp130 (bold). From N- to C-terminus mCherryVHH-gp130: signal peptide of IL-11R (blue), FLAGG-tag (green), HA-tag (purple), mCherryVHH (red) and 13 aa of the ECD, TM and ICD gp130 (bold). (B) Amino acid sequences of synthetic cytokines GFP-Fc, mCherry-Fc, GFP-mCherry-Fc and GFP-GFP-mCherry-Fc fusion proteins. From N- to C-terminus: signal peptide of IL-11R (blue), FLAGG-tag (red), GFP (green) or mCherry (purple) or both, TEV protease cleavage side (bold) and the Fc domain (orange). (C). Amino acid sequences of synthetic cytokines mCherry3 and GFP3. From N- to C-terminus: mCherry (red), GFP (green) trimeric GCNpII motif (brown), 6xHis tag (purple), myc tag (blue). (TIFF) [file pone.0230804.s001.tiff]

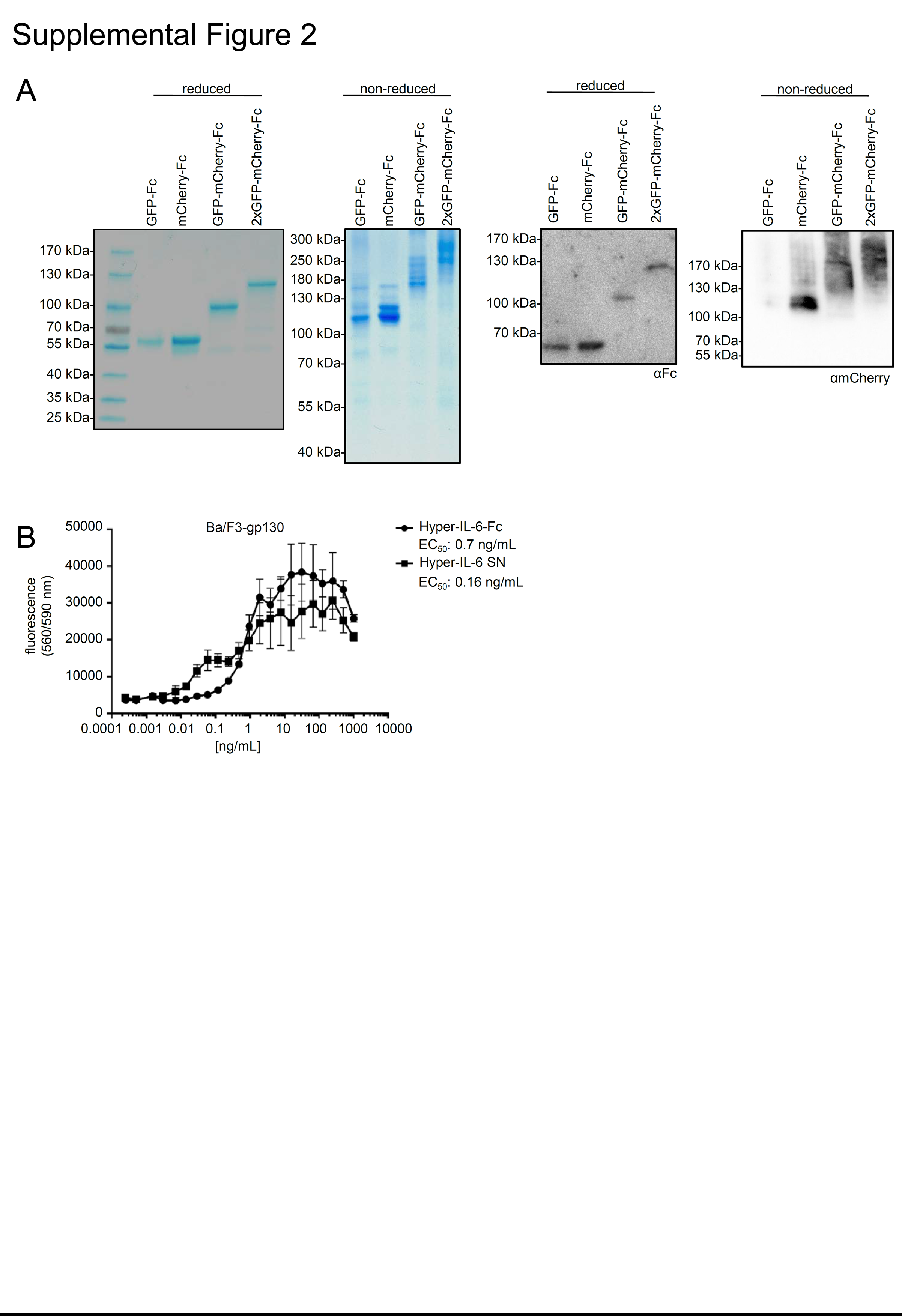

Supplement: S2 Fig — (A) Coomassie gel (left) and Western blot (right) analysis with anti-Fc and anti-mCherry of all purified proteins under reducing and non-reducing conditions. For Coomassie staining 5 μg, for western blot 5 ng of protein were loaded. (B) Proliferation of Ba/F3-gp130 cells with increasing concentrations of 0.0004–1000 ng/ml purified Hyper-IL-6-Fc or Hyper-IL-6 from CHO-K1 cell culture supernatants. One representative experiment out of three is shown. (TIFF) [file pone.0230804.s002.tiff]

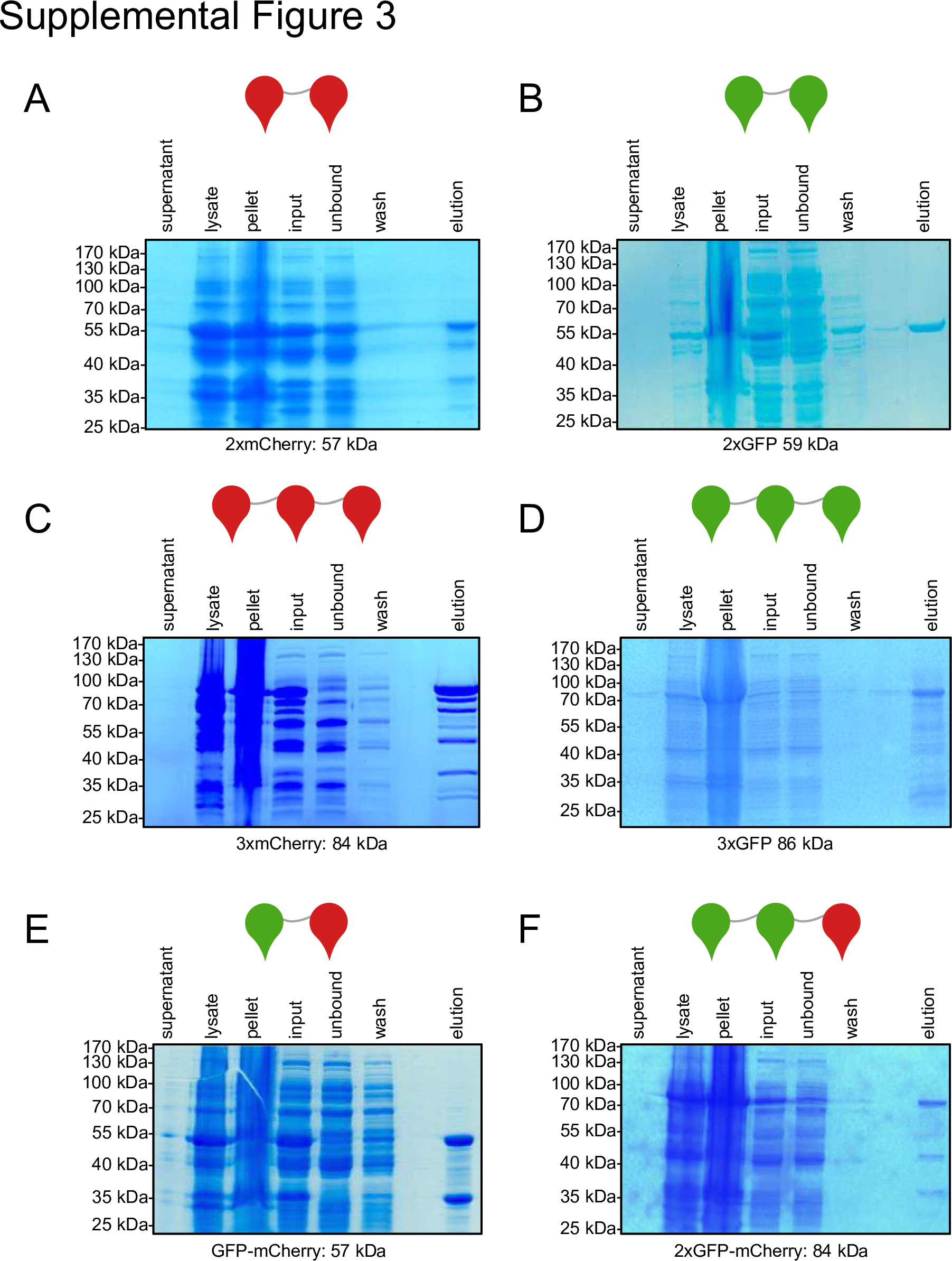

Supplement: S3 Fig — Coomassie staining of the purification procedure of (A) 2xmCherry, (B) 2xGFP, (C) 3xmCherry, (D) 3xGFP, (E) GFP-mCherry and (F) GFP-GFP-mCherry proteins expressed in E. coli. The image is similar to the original image and for illustrative purposes only. (TIF) [file pone.0230804.s003.tif]

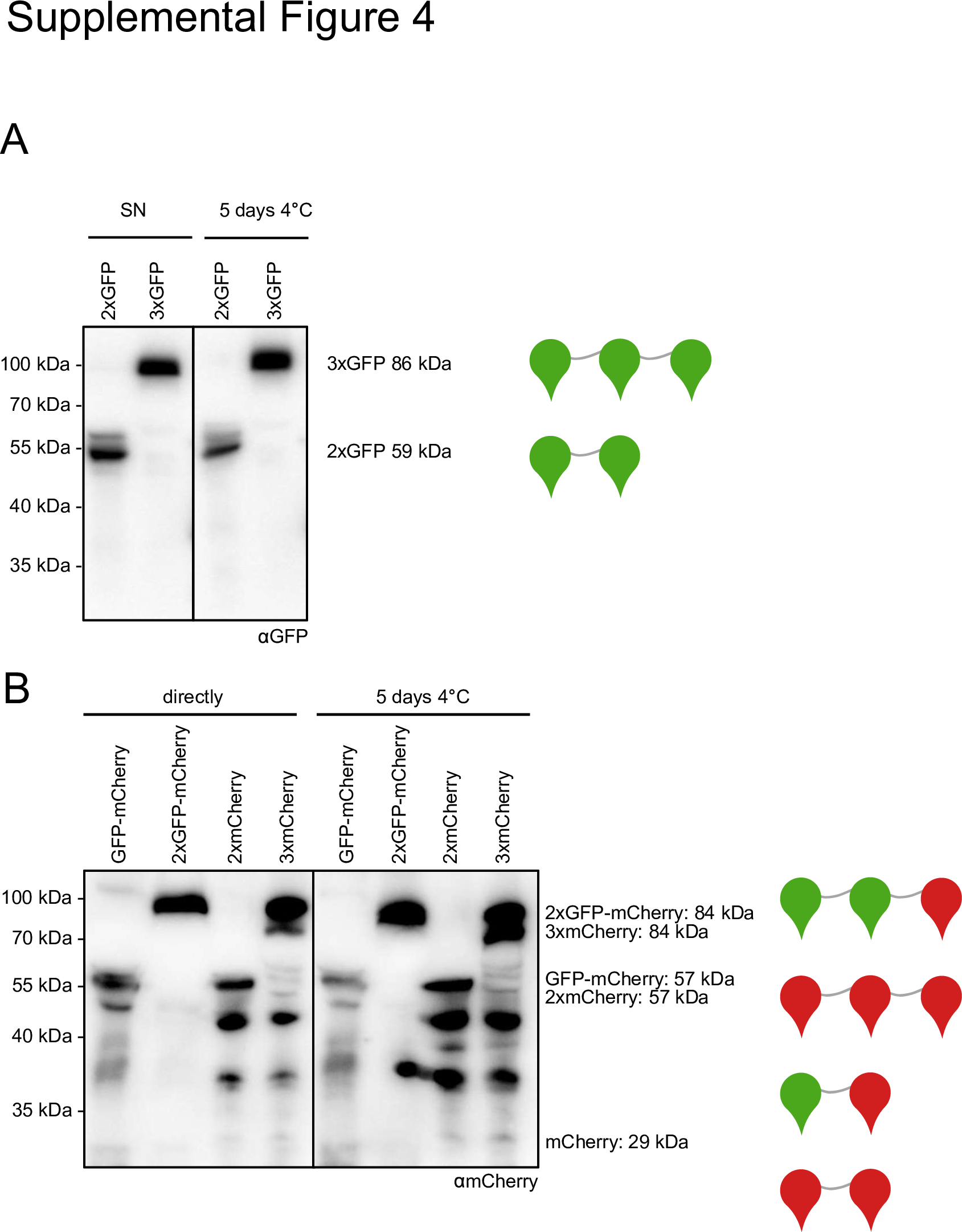

Supplement: S4 Fig — (A) CHO-K1 cells were stably transduced with the respective cDNA coding for 2xGFP, 3xGFP. The supernatants were collected and expression analyzed via western blot using anti-GFP antibodies. The respective supernatants were either analyzed directly or after storage at 4°C for 5 d. The image is similar to the original image and for illustrative purposes only. (B) CHO-K1 cells were stably transduced with the respective cDNA coding for GFP-mCherry, GFP-GFP-mCherry, 2xmCherry and 3xmCherry. The supernatants were collected and the expression analyzed via western blot using anti-mCherry antibodies. The respective supernatants were either analyzed directly or after storage at 4°C for 5 d. The image is similar to the original image and for illustrative purposes only. (TIF) [file pone.0230804.s004.tif]
