# Supplementary material for: Multimerization strategies for efficient production and purification of highly active synthetic cytokine receptor ligands
Source: PLoS One. 2020 Apr 1;15(4):e0230804. doi: 10.1371/journal.pone.0230804 (PMC7112226; doi:10.1371/journal.pone.0230804)
Supplement: Attachment — Submitted filename: Response to Reviewers.docx [file pone.0230804.s005.docx]

**Reviewers' Comments to Author:**

**Reviewer 1:**

I think the authors have made the point that different synthetic heterodimeric or heterotrimeric can be used for receptor activation.

I suggest the authors to add a "conclusion" to the manuscript to allow the reader to have a short take home message and a look into the future.

Some spelling mistakes are in the ms, maybe a native English speaker should go through the text before publication.

**Author´s answer:**

We have carefully revised the manuscript and have corrected spelling mistakes.

Additionally, we have added a “Conclusion”-section at the end of the manuscript.
